# Wool-Like Hollow Polymeric Nanoparticles for CML Chemo-Combinatorial Therapy

**DOI:** 10.3390/pharmaceutics10020052

**Published:** 2018-04-18

**Authors:** Barbara Cortese, Stefania D’Amone, Ilaria Elena Palamà

**Affiliations:** 1 Nanotechnology Institute, CNR-Nanotechnology Institute (CNR-NANOTEC), University La Sapienza, P.zle A. Moro, 00185 Roma, Italy; barbara.cortese@nanotec.cnr.it; 2 Nanotechnology Institute, CNR-Nanotechnology Institute (CNR-NANOTEC), Monteroni street, 73100 Lecce, Italy; stefania.damone@nanotec.cnr.it

**Keywords:** combinatorial therapy, polymeric nanoparticles, CML cells

## Abstract

Chronic myeloid leukaemia (CML) is caused by the BCR-ABL oncogene, which encodes the constitutively active BCR-ABL tyrosine kinase. Targeted therapy with tyrosine-kinase inhibitors induces a partial cytogenetic response in most patients. Nanosystems can represent an opportunity for combinatorial therapy with the capacity to simultaneously release different therapeutic agents, checking the pharmacokinetic properties. In this work, we have developed a novel poly-(ε-caprolactone) (PCL) nanosystem for combinatorial therapy in CML, composed of a biodegradable pH sensitive core releasing Nilotinib (Nil) and an enzymatic sensitive outer shell releasing Imatinib Mesylate (IM), resulting in wool-like nanoparticles (NPs). The resulting double loaded wool-like hollow PCL NPs showed a high dual-drug encapsulation efficiency, pH and enzymatic sensitivity and synchronized drug release capability. The combinatorial delivery of IM and Nil exhibited an importantly reduced IC_50_ value of IM and Nil on leukaemia cells compared to single free drugs administration. In vitro results, showed that combinatorial nanomixures preserved the biological activity of loaded drugs for extensive time windows and led to a constant release of active drug. In addition, the combination of IM and Nil in single PCL NPs have shown a more therapeutic efficiency at a low dose with respect to the single drug nanomixures, confirming that both drugs reached the target cell precisely, maximizing the cytotoxicity while minimizing the chances of cell resistance to drugs.

## 1. Introduction

Chronic myeloid leukaemia (CML) is a myeloproliferative disorder, linked with a constitutively active tyrosine kinase BCR-ABL [1]. Discovery of Tyrosine Kinase Inhibitors (TKIs), such as Imatinib Mesylate (IM), has resulted in meaningfully enhanced survival and disease management in CML patients [2]. Yet, a population of several subclones, that is, resistant-IM have been found [3,4,5,6]. These subclone cells have shown to be unresponsive to TKI therapy and are leading to disease relapse on discontinuation of drug treatment [7]. In many patients, resistance is due to BCR-ABL tyrosine kinase activity reactivation caused by the presence of point mutations within numerous regions of the Abl kinase domain [8,9]. Such mutations harm IM binding by affecting interaction residues or by making a BCR-ABL conformation to which IM is incapable of binding. More than 40 dissimilar point mutations (such as M244V, G250E, M35IT, E255V, Q252H, etc.), encoding for different amino acid changes in the Bcr-Abl kinase domain, have been observed in relapsed CML patients [10]. Consequently, second generation inhibitors may address TKI resistance, which comprise Nilotinib (Nil) [11], derived from IM with ~30-fold higher potency. In addition, Nil like IM, binds the inactive conformation of Bcr-Abl. In particular, the mutation M351I is more sensitive to Nil but does not inhibit T315I mutation as well as IM and Dasatinib (Das) [12,13].

Recently, clinical observation has revealed that co-administration of IM and other TKIs, such as Nil [14,15] or Dasatinib [16], may offer an additive/synergistic anti-leukaemia effect. It has been shown that IM affects malignant cells, while other TKIs have a critical role in inhibiting or eradicating the several subclones, that is, resistant-IM cells [14,15]. In addition, malignant clones associated with resistance to other TKIs may be susceptible to IM, thus reducing the whole resistance probabilities. Additive/synergistic anti-proliferative properties from a TKIs combination could lead to the eradication of a higher percentage of residual leukemic cells. Combinatorial therapy strategies that potentiate single drugs and overcome adverse toxicity may not only have important therapeutic and pharmacological applications but also represent a radical cure for CML.

Targeting cancer cells is especially relevant for avoiding serious side-effects on healthy cells caused by unspecific action of anticancer drugs. However, existing treatments are restricted by the dissimilar pharmacokinetics and biodistribution of different drugs and present limited elasticity for management optimization. The challenge in developing new therapies for CML lies in the successful combination of therapeutic and targeting actions into one system.

Combinatorial therapy is one of most promising approaches in CML chemo-treatment. To enhance their effects, drugs should be used at their best concentrations at different times, resulting in an additive or synergistic therapeutic effect. In addition, to supply the single therapeutic agent with an optimal dose, the release performances of each active agent in a co-delivery nanosystem should be controlled independently. In fact, when several active therapeutic molecules are carried out in single systems, the synergistic properties can be envisioned for CML therapy and can prevent drug resistance, because of their capacity to adjust to the optimal drug dose at the cell target [17,18]. In addition, co-delivery nanosystems can stimulate drug synergism carrying several active agents to the target sites at the same time and can represent an alternative approach to creating new fitting chemotherapeutics [19].

Nanocarriers for the co-delivery of many active agents have been extensively studied [20,21,22,23,24]. Multidrug delivery systems with different release behaviours are more problematical to formulate. Numerous approaches have been exploited to co-deliver many therapeutic molecules into a single system, such as physical packing into core particles [25,26], chemical conjugation [17,18] and covalent bonds between polymer and drugs [27].

Polymer-based nanoparticles can be efficiently tailored with large molecules such as proteins, or with small molecule entrapment such as nucleic acids, to extend drug circulation half-life, decrease non-specific uptake and for advanced delivery control by targeting the tumours’ site [28,29]. Moreover, the polymers’ features, such as cross-linking and polyionic complexation, can improve the product’s loading efficacy and release kinetics [30,31,32]. Physical loading is an encouraging drug loading stratagem that could offer multiple active agent delivery with a different release level for each molecule encapsulated.

The release degree can be tuned by changing the quantity of the payload incorporated as well as polymer degradation time.

Multi-layered microcapsules can also allow simultaneous multiple drug delivery [33,34]. In this context, encapsulation of different TKI molecules in a single stimuli responsive polymeric nanosystem can be indispensable for killing resistant CML cells.

Our recent reports have defined that IM loaded polymeric nanoparticles can show sustained drug delivery, reducing cytotoxicity against healthy cells and increasing the drug concentration in target cells [35,36,37].

In this study, we explore an approach to achieve the simultaneous delivery of two TKI molecules, IM and Nil, using a single polymeric nanoparticle responsive to pH and intracellular protease action, to promote their synergistic anti-CML activity.

Recently, Food and Drug Administration (FDA)-approved poly(ε-caprolactone) (PCL) has been used for the synthesis of stimuli-responsive NPs. A notable aspect is its biodegradability, biocompatibility and drug permeability, as well as its low cost if compared to other biodegradable polyesters.

Specifically, in this work, the hydrophilic core was loaded with Nil complexed with pH-sensitive chitosan (CH) polymer. Loaded drugs are typically released through diffusion and/or polymer degradation [38,39]. As a result, there may be some lag from administration to reaching the therapeutic threshold. To achieve a quicker release, we have incorporated in the PCL NP core a mixture of sodium bicarbonate and potassium tartrate (NaHCO_3_ + KC_4_H_5_O_6_), which quickly generates CO_2_ bubbles in an acid environment, producing large pores in the NP shell and burst release of the contents (sparkling NPs). A layer of IM complex with a protease-sensitive dextran (DXS) forms an outer shell of sparkling NPs, giving wool-like NPs. In this way, we have obtained a 2-step release mechanism of stimuli-responsive NPs: (i) IM will be released in the cytoplasm from IM-DXS complexes thanks to intracellular protease (first release) and (ii) Nil-CH complexes will be released in the lysosomal compartment following the generation of CO_2_ bubbles (second release). In this way, the first release of IM can rapidly induce a partial inactivation of BCR-ABL oncoprotein and subsequently the second release of Nil can determinate the complete oncoprotein inactivation, reducing resistance probabilities.

Additionally, our wool-like PCL NPs present a negative charge in physiological pH, which reverses to a positive charge in the acidic pH typically present in endo-lysosomal vesicles. This behaviour determines the endo-lysosomal escape of our NPs, which is crucial for enhancing the drug efficacy. We further analysed the synergistic therapeutic effect of a new stimuli responsive co-delivery system of the wool-like PCL NPs, on CML cells.

In vitro studies showed an exceptional anti-leukemic activity of wool-like IM/Nil PCL loaded NPs on the KU812 model cell line. Combining the pH sensibility of the core and the enzyme sensitivity of the outer shell improved the drug’s kinetics and efficacy on cell death and on the down-regulation of BCR-ABL oncoprotein. To the best of the authors’ knowledge, it is the first time that three-dimensional wool-like and sparkling nanoparticles have been synthesized. Such a drug delivery system can have important therapeutic and pharmacological applications.

## 2. Materials and Methods

### 2.1. Material

All tissue culture media and serum were purchased from Sigma-Aldrich and cell lines were purchased from the American Tissue Type Collection (ATTC) (Manassas, VA, USA). The following were supplied by Sigma-Aldrich (Saint Louis, MO, USA ): thiazolyl blue tetrazolium bromide (MTT), dextran sulphate (DXS), phosphate buffered saline (PBS), Fluoroshield with DAPI, chitosan (CH) of low molecular range with a degree of deacetylation of 75–85%, poly-(ε-caprolactone) (PCL) with an average molecular weight (MW) of 14,800 Da and polyvinyl alcohol (PVA, MW 13–23 kDa, 87–80% hydrolysed), potassium sodium tartrate tetrahydrate, 3,3′-Dioctadecyloxacarbocyanine perchlorate (DiO), anti-clathrin light chain monoclonal antibody. LysoTracker was from Life Technology (Carlsbad, CA, USA). Nilotinib, anti BCR antibody, AnnexinV-PI kit by Abcam (Cambridge, UK).

### 2.2. Synthesis and Characterization of Sparkling and Wool-Like PCL NPs

Sparkling and wool-like hollow PCL NPs were obtained by an emulsion-diffusion-evaporation modified method [37,40,41]. Briefly, sparkling Nil-PCL NPs were obtained as follows: a solution of Nilotinib (10 to 100 μM) was complexed with 0.5% (*w*/*v*) chitosan in an acetic acid solution by incubating at RT for 24 h on a rotary shaker. A 2.5 mg of potassium sodium tartrate tetrahydrate and Nil-CH complexes (10 to 100 nM) were added to 1 mL of aqueous solution of PVA (10 mg/mL) and mixed in ice for 10 min. After, the mix was emulsified with 2 mL of PCL (5 mg/mL) in chloroform to obtain the first water/oil (W/O) emulsion. This W/O emulsion was kept under constant agitation on a magnetic stirrer at 1500 rpm for 10 min in ice. Subsequently, 6 mL of PVA (10 mg/mL) was added to the first emulsion and incubated for 75 min under constant stirring (1500 rpm) in ice to form the second W/O/W emulsion. 15 mL of deionized water was added in order to remove chloroform and solidify the PCL NPs. The mix was kept under constant stirring (1500 rpm) for 1 h. Next, the NPs suspension was washed three times with water by centrifugation at 3500 rpm for 20 min and then suspended in water and stored at 4 °C until use. Wool-like hollow Nil/IM PCL NPs were obtained by coating with the Layer by Layer technique (LbL), the sparkling PCL NPs using a DXS loaded IM solution. Prior to LbL coating, DXS (2 mg/mL in 0.1 M NaCl, pH 6.5) was premixed with IM (10 to 100 μM) under agitation overnight at Room Temperature (RT). The mixture was then dialyzed with pure water for 8 h. For fluorescent PCL NPs, a 0.05 mg/mL of DiO in chloroform was added to PCL solution and the preparation was carried out as described before. The labeled nanoparticles were stored in the dark at 4 °C until use.

The average particle size and zeta potential of sparkling and wool-like PCL NPs were evaluated by photon correlation spectroscopy employing a Zetasizer Nano ZS90 (Malvern Instruments Ltd., Royston, England) supplied with a 4.0 mW He-Ne laser working at 633 nm and a photodiode detector. Measurements were performed at 25 °C in aqueous solutions (pH 7.4).

The stability of wool-like PCL NPs was verified under physiological environments. NPs were hatched in complete Roswell Park Memorial Institute (RPMI) medium at 37 °C and the size variation was measured over a period of 8 days by Dynamic Light Scattering (DLS) analysis. Measurements were taken from three independent experiments, conducted in triplicate (Student’s *t*-test, *P* < 0.05).

The external morphology of the sparkling and wool-like PCL NPs was examined by scanning electron microscopy (SEM). Prior to SEM analysis, the samples were coated with a 10 nm gold layer. SEM analyses were taken with a Carl Zeiss Merlin SEM supplied with a Gemini II column and a Field Emission Gun (FEG).

### 2.3. Drugs Entrapment Efficacy and *In vitro* Release

The drug entrapment efficacy of sparkling and wool-like PCL NPs was evaluated by examining the final emulsion supernatant after the centrifugation of NPs. Drugs content present in the supernatant (100 μL of supernatant diluted in 1 mL of PBS 1× pH 7.4) was determined by UV-visible spectrophotometer (Varian Cary^®^ 300 Scan; Varian Instruments, Palo Alto, CA, USA) at a wavelength of 270 nm (for Nil) and 260 nm (for IM). The supernatant concentration was calculated from the UV-visible adsorption referring to a standard curve. The encapsulation efficacy of Nil/IM in PCL NPs was calculated using the following Equation (1):

(1) $$Encapsulation\;efficacy\;\left(\%\right)=\frac{\left(mass\;of\;the\;drug\;in\;NPs\right)}{\left(Initial\;mass\;of\;drug\;used\right)}\times \;100$$

In vitro release of Nil/IM PCL NPs was examined by dialysis method. 1 mL of PCL NPs (10 mg/mL) was placed in dialysis bags (MWCO 8 kDa to 12 kDa) in 100 mL of PBS 1× (pH 5.0, 6.5 and 7.4) with 5% Tween 80 and stirred at 100 rpm at 37 °C. At various time intervals (0 to 96 h), 1 mL of the dialysis medium was analysed by UV-visible spectrophotometer; same volume of fresh PBS 1× was added. Accumulated release percentage of drugs was determined by Equation (2):

(2) $$Release\;percentage\;\left(\%\right)=\frac{W_{\mathrm{release}}}{W_{\mathrm{total}}}\times \;100$$

where *W*_release_ was the quantity of Nil or IM released from PCL NPs into dialysis bag at different times and *W*_total_ was the total quantity of Nil and IM in PCL NPs.

Measurements were taken from three independent experiments, conducted in triplicate (Student’s *t*-test, *P* < 0.05).

### 2.4. Wool-Like PCL NPs Cellular Uptake and Intracellular Localization

Human chronic myeloid leukaemia cells (KU812) and human normal B lymphoblast (C13589) were cultured in RPMI 1640 supplemented with 10% fetal bovine serum at 37 °C in 5% CO_2_ in a humidified incubator. Fluorescent DiO NPs were used as a probe to analyse the cellular uptake of blank PCL NPs, IM-PCL NPs, sparkling Nil-PCL NPs and wool-like Nil/IM PCL NPs. KU812 cells at log phase (10^5^ cells) were treated with serum-free RPMI medium containing fluorescent DiO wool-like PCL NPs (0.05 mg/mL) and incubated for 0–4 h at 37 °C in CO_2_ incubator. Successively, the cells were washed three times with cold PBS 1×, pH 7.4. Subsequently, all the wells were lysed by 0.5% Triton X-100 in a 0.2 N NaOH solution. The amount of DiO (excitation wavelength of 484 nm and an emission wavelength of 501 nm) in the cells was fluorometrically evaluated for the lysate using a fluorescence spectrometer. The cellular uptake efficiency (%) was indicated as the fluorescence associated with the cells versus the ones present in the positive control solution. Representative measurements of three distinct sets of data have been reported (Student’s *t*-test, *P* < 0.05).

For fluorescence confocal analysis, cells were fixed in situ for 5 min in 3.7% formaldehyde and mounted with fluoroshield with DAPI. Intracellular localization of DiO wool-like PCL NPs after 1 h of incubation were studied with LysoTracker Red (Life technology) immunostaining, according to the manufacturer’s instructions, to label lysosomes. The uptake modality of DiO wool-like PCL NPs after 1 h was evaluated with anti-clathrin light chain monoclonal antibody (10 μg/mL; Sigma-Aldrich, Saint Louis, MO, USA) immunostaining according to the manufacturer’s instructions. The primary antibody was revealed using TRITC conjugated anti-mouse antibody (4 μg/mL; Millipore, Burlington, MA, USA) as secondary antibody and mounting with fluoroshield with DAPI.

Confocal micrographs were taken with a Leica confocal scanning system mounted on a Leica TCS SP5 (Leica Microsystem GmbH, Mannheim, Germany), using a 40× and 63× oil immersion objective and spatial resolution of 200 nm in x-y and 100 nm in z.

### 2.5. Quantitative Study of Co-Localization

Quantitative co-localization analysis of ten fields, within three different experiments, were casually selected for each sample. As previously reported [37], coefficient of correlation (CC or Pearson’s *r*) [42], intensity correlation quotient (ICQ) [43] and overlap coefficient (OC) [44] as frequency indices of co-localization between NPs and LysoTracker were used. Coefficients and quotients were calculated from each confocal image, as well as pixel shuffling and calculation of the 999⁄1000 quantile of intensity, were conducted with custom-made plug-in programs (available at http://www.mbs.med.kyoto-u.ac.jp/imagej/index.html) combined with ImageJ software (National Institute for Health, Bethesda, Rockville, MD, USA). Numerical data was processed with Excel (Microsoft, Redmond, WA, USA) for further calculation. Representative measurements of three distinct sets of data have been reported (Student *t*-test, *P* < 0.05).

### 2.6. In Vitro Combinatorial Anti-Leukaemia Efficacy

Synergistic assay. In vitro cytotoxicity of blank PCL NPs, free IM, free Nil, free combination of IM and Nil, IM-PCL NPs, sparkling Nil-PCL NPs, wool-like Nil/IM PCL NPs against human chronic myeloid leukaemia cells (KU812) and healthy C13895 cells was evaluated via MTT assay after 48 h of incubation, according to the manufacturer’s instructions (Sigma-Aldrich, Saint Louis, MO, USA). The IC50 of IM, Nil and free combination was calculated. The absorbance was spectrophotometrically measured at a wavelength of 570 nm and the background absorbance measured at 690 nm was subtracted. The percentage viability was expressed as the relative growth rate (*RGR*) by Equation (3):

(3) $$RGR\;\left(\%\right)=\frac{D_{\mathrm{sample}}}{D_{\mathrm{control}}}\;\times 100$$

where *D*_sample_ and *D*_control_ were the absorbance of the sample and the negative control. Each experiment was repeated three times in triplicate (Student’s *t*-test, *P* < 0.05).

Synergism was examined by determining the Nil and IM combination index (CI) with the isobologram equation of Chou and Talalay [45]. The combination index was expressed by Equation (4):

(4) $$CI=\frac{a}{A}\;+\;\frac{b}{B}$$

where, *a* was the Nil at concentration IC_50_ in combination with IM at concentration *b*, while *A* was the Nil IC_50_ and *B* is the IM IC_50_. A synergistic effect was observed when the *CI* < 1. An additive effect was observed when *CI* = 1 and antagonistic effect was observed when *CI* > 1.

Apoptosis and cell cycle analysis. Cell apoptosis and DNA distribution in cell cycle were analysed by flow cytometry. Briefly, 10^5^ KU812 leukaemia cells were treated with blank PCL NPs (0.05 mg/mL), free IM (150 nM), free Nil (30 nM), free combination of IM (130 nM) and Nil (28 nM), IM-PCL NPs (70 nM), sparkling Nil-PCL NPs (18 nM), wool-like Nil/IM PCL NPs (Nil 15 nM, IM 50 nM) for 24 h at 37 °C, 5% CO_2_. Only RPMI medium was used as control. After incubation, KU812 cells were washed with PBS 1× and staining with Annexin V-FITC/PI according to the manufacturer’s instructions (Abcam). Cell apoptosis and cell cycle distribution were determined by analysing 10,000 ungated cells using a Flow Cytometer (C6, Accuri, Ann Arbor, MI, USA). All experiments were performed in triplicate (Student’s *t*-test, *P* < 0.05).

Analysis of oncoprotein BCR-ABL cellular pattern distribution. Combinatorial therapeutic effect of Nil/IM wool-like PCL NPs was investigated on cellular pattern expression of oncoprotein BCR-ABL. KU812 human chronic myeloid leukaemia (10^5^ cells) were treated with serum-free RPMI medium containing blank PCL NPs (0.05 mg/mL), free IM (150 nM), free Nil (30 nM), free combination of IM (130 nM) and Nil (28 nM), IM-PCL NPs (70 nM), sparkling Nil-PCL NPs (18 nM), wool-like Nil/IM PCL NPs (Nil 15 nM, IM 50 nM) and cultured for 24 h at 37 °C in CO_2_ incubator. Cells were washed three times with PBS 1× and fixed in 3.7% formaldehyde for 15 min. After the cells were permeabilized for 5 min with perm buffer (pH 7.2) composed of PBS 1× with 10% sucrose, 0.3% NaCl, 0.4% Hepes and 0.5% Triton X-100. Successively, the cells were incubated for 5 min with 1% bovine serum albumin in PBS 1× to block non-specific binding. They were then incubated with anti-Bcr antibody (10 μg/mL; Abcam) at 37 °C for 1 h. The primary antibody was revealed using TRITC conjugated anti-mouse antibody (4 μg/mL; Millipore) as secondary antibody and mounted with DAPI. Confocal micrographs were taken with a Leica confocal scanning system mounted on a Leica TCS SP5 (Leica Microsystem GmbH, Mannheim, Germany), furnished with a 63× oil immersion objective and spatial resolution of 200 nm in x-y and 100 nm in z.

### 2.7. Statistical Analysis

Statistical analyses were achieved using a Student’s *t*-test. The differences were considered significant for *P* values < 0.05.

## 3. Results and Discussion

### 3.1. Preparation and Physico-Chemical Characterization of Drug Loaded Sparkling and Wool-Like Hollow PCL NPs

**Scheme 1:**
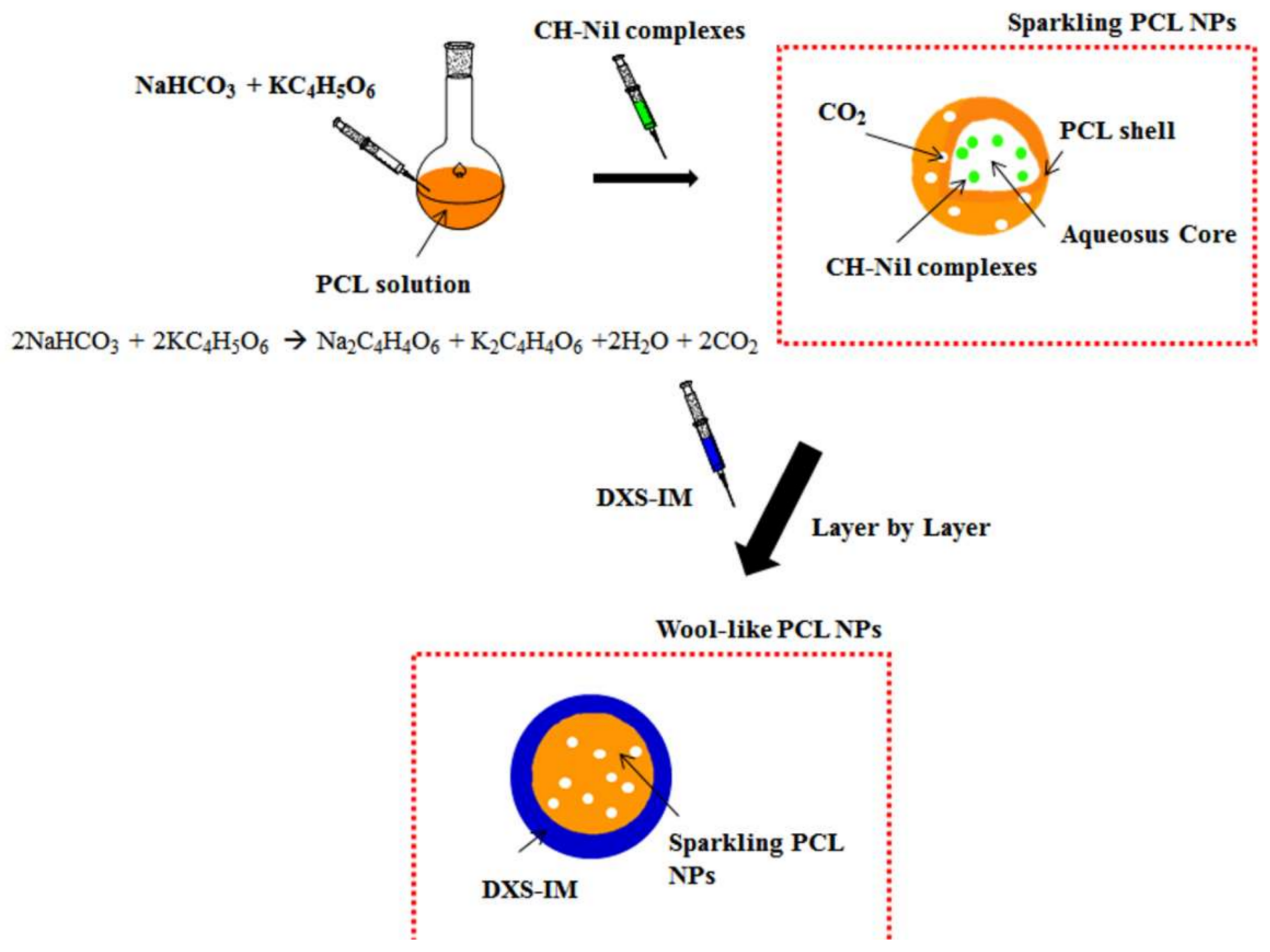
Schematic illustration of the production of sparkling Nilotinib (Nil) loaded poly-(ε-caprolactone) (PCL) nanoparticles (NPs) and the wool-like hollow Nil/ Imatinib Mesylate (IM) loaded PCL NPs.

**Figure 1:**
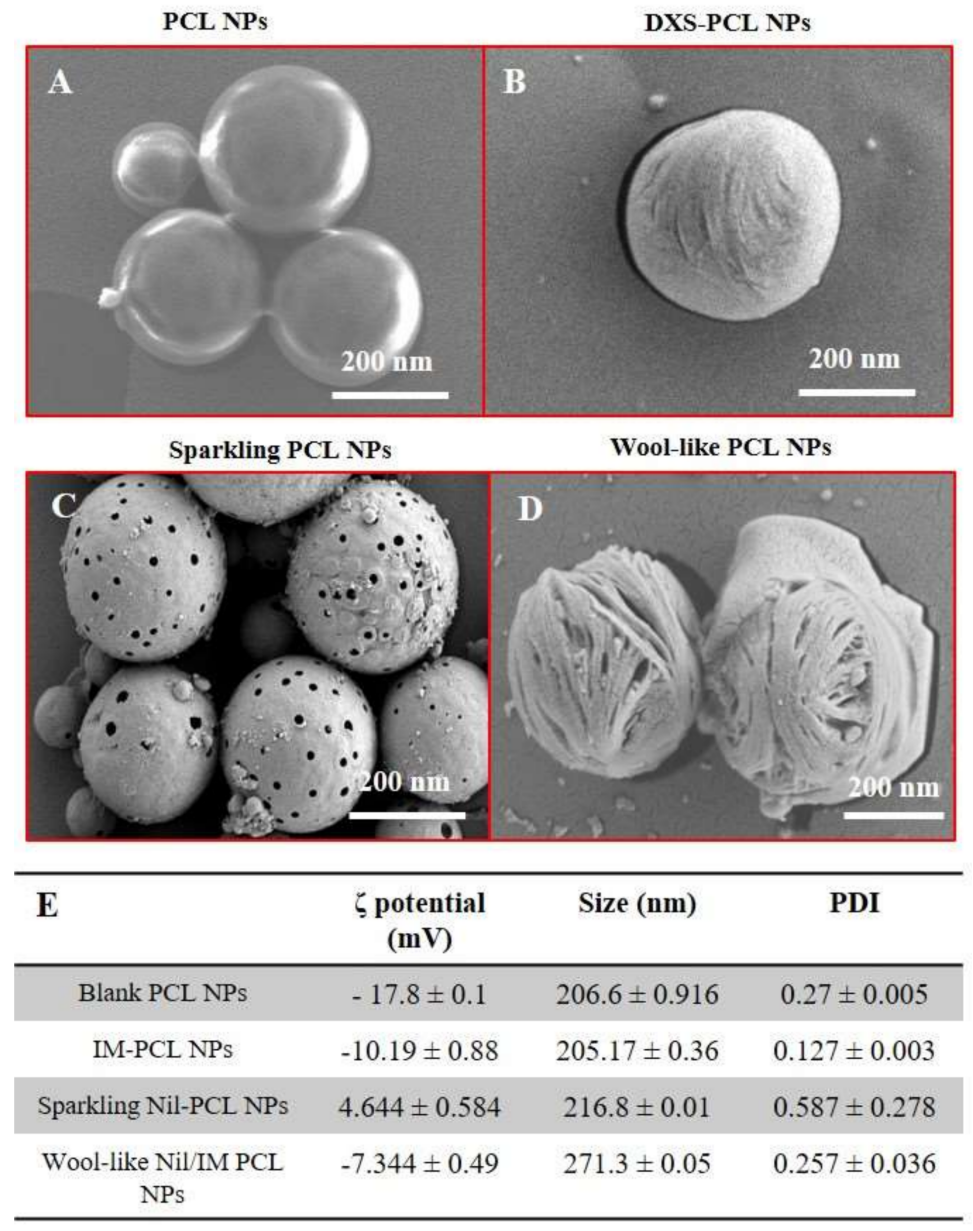
Scanning electron microscopy (SEM) images of blank PCL NPs (**A**), IM-DXS loaded PCL NPs (**B**), burst sparkling Nil loaded PCL NPs (**C**) incubated in acid condition (pH 5.0) and wool-like hollow Nil/IM loaded PCL NPs (**D**). Magnification: 34.01 KX. Scale bars: 200 nm. Physicochemical characterization of different PCL NPs formulation (**E**). Representative measurements of three distinct sets of data have been reported (Student’s *t*-test, *P*-values < 0.05).

Preparation of the investigated co-delivery system is schematically shown in Scheme 1 and a SEM image of the nanoparticles is shown in Figure 1.

Sparkling and wool-like hollow PCL NPs were synthetized in highly reproducible manner by a modified emulsion-diffusion-solvent evaporation method [37,40,41]. In particular, sparkling Nil loaded PCL NPs present a PCL shell and an aqueous core with Nil complexed with pH-sensitive Chitosan (CH) and a mixture of sodium bicarbonate and potassium tartrate (NaHCO_3_ + KC_4_H_5_O_6_) which quickly generates CO_2_ bubbles in an acid environment, producing large pores in the NP shell and burst release of Nil contents. Using the LbL technique, the outer shell of sparkling Nil PCL NPs was coated with a layer of IM complexed with a protease-sensitive dextran (DXS), obtained a wool-like polymeric PCL NPs. Our co-delivery system allows to formulate a combinatorial therapy in a single nanoparticle and, at the same time, to transport them to leukaemia cells.

SEM images of blank PCL NPs, IM-PCL NPs, sparkling Nil-PCL NPs, wool-like Nil/IM PCL Ps (Figure 1A–D) reveals that the NPs have a spherical morphology and the size was in agreement with DLS data (Figure 1E). Sparkling Nil-PCL NPs have showed an average hydrodynamic size of 216.8 ± 0.01 nm, which was smaller than the wool-like Nil/IM PCL NPs (271.3 ± 0.05 nm). Small nanoparticles are potentially preferable for drug delivery for a better collection at oncogenic sites, using the enhanced permeation and retention (EPR) effect [46].

Different surface morphology between the sparkling Nil-PCL NPs (Figure 1C) and the wool-like Nil/IM PCL NPs was observed by SEM analysis (Figure 1D). In particular, the sparkling Nil-PCL NPs presented large pores when incubated in acid conditions (pH 5.0), while the wool-like Nil/IM PCL NPs showed a surface that reminded a ball of wool due to the DXS coating. The nanoparticles could be lyophilized without any adjuvants and the resuspended NPs were stable and uniform. In addition, the stability of drug-loaded PCL NPs was verified in physiological conditions. NPs were incubated in complete RPMI medium at 37 °C for a time window of 8 days and the size was measured by DLS. Drug-loaded PCL NPs over a period of 8 days maintain their hydrodynamic size and PDI (data not shown), supporting NPs stability in physiological conditions.

Surface modification of the different PCL NPs can be easily followed by zeta-potential measurements, as show in Figure 1E. The negative surface charge of wool-like Nil/IM PCL NPs (−7.344 ± 0.49 mV) might contribute to improve blood compatibility and protract NPs circulation time decreasing the RES clearance. In addition, changes in the physico-chemical characteristics of wool-like PCL NPs surface after cellular uptake, such as the release of the IM by intracellular protease degradation of DXS (first release) and the cationization of sparkling Nil-PCL NPs (4.644 ± 0.584 mV) in acid environment, influenced the escape from the endo-lysosomal compartment of NPs, after the generation of CO_2_ bubbles producing the large pores in the PCL shell.

**Figure 2:**
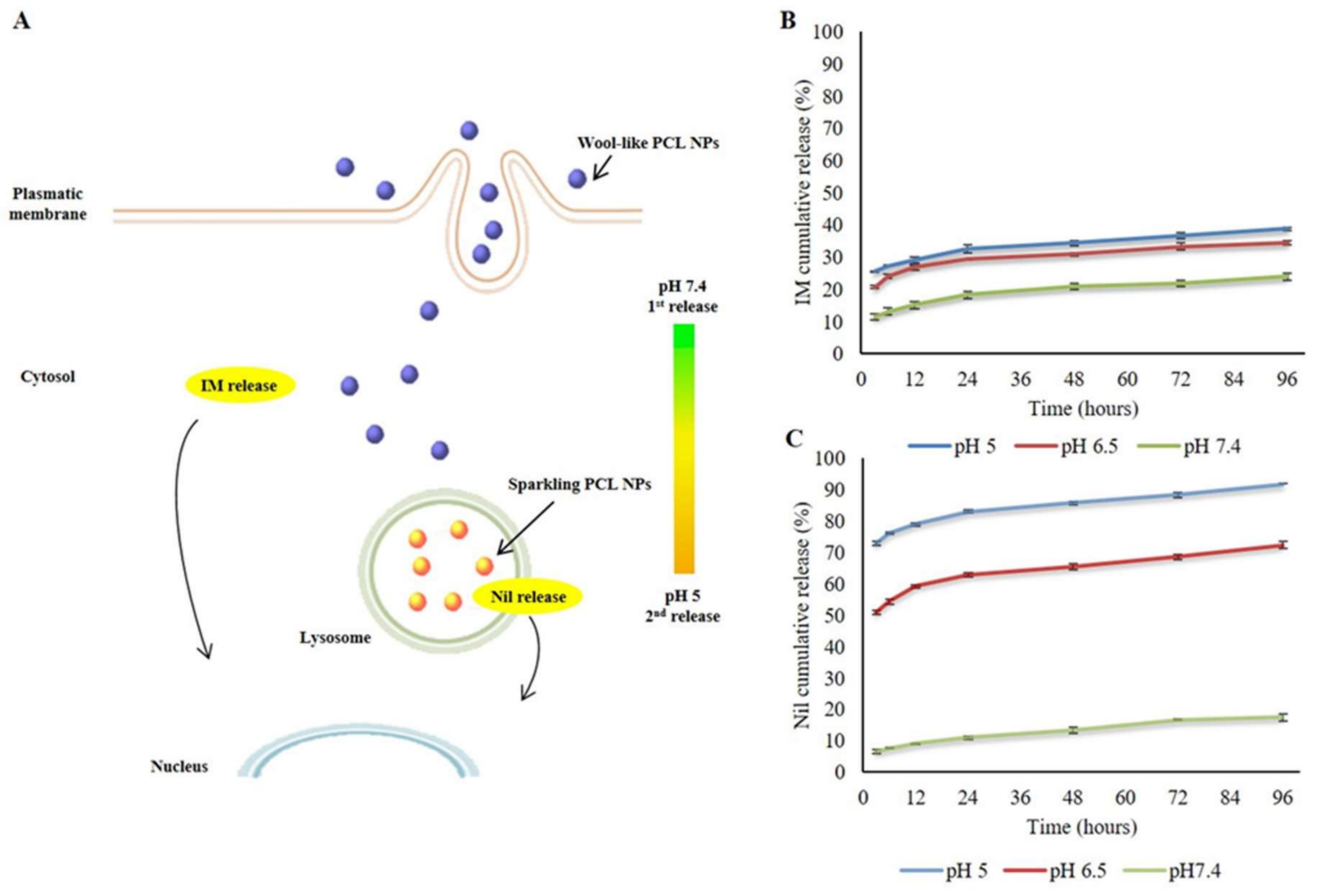
Expected mechanism of intracellular trafficking and release of the IM and Nil from the stimuli responsive PCL NPs (**A**). In vitro drug cumulative release of IM (**B**) and Nil (**C**) from wool-like IM/Nil loaded PCL NPs at 37 °C under neutral conditions (pH 7.4) and acidic conditions (pH 6.5 and 5.0). Representative measurements of three distinct sets of data have been reported no significant difference between values at different time points (Student’s *t*-test, *P* < 0.05).

A schematization of the expected intracellular trafficking and 2-step release mechanism of wool-like hollow PCL NPs is depicted in Figure 2A: the first step shows the release of IM in the cytoplasm from IM-dextran complexes thanks to intracellular protease (first release); within the second step Nil-CH complexes were released in the lysosomal compartment (pH 5.0) following the generation of CO_2_ bubbles (second release). A successful nanosystem should have a high loading capability to decrease the amount of the therapeutic agents necessary for the therapy [47]. Moreover, drug loading in polymeric NPs offers a continuous release for a longer time windows and protection by degradation [48]. In our study the TKI molecules (IM and Nil) were proficiently loaded in the wool-like PCL NPs with great encapsulation efficiency. In particular, the encapsulation efficiency percentage (EE%) of wool-like of Nil/IM PCL NPs was 55.26% for Nil and 95.23% for IM. The co-loading process did not reduce the EE% of IM relative to IM-PCL NPs (~95.2%) and the EE% of Nil in relation to sparkling Nil-PCL NPs, wool-like Nil/IM PCL (~55%). These data suggested that the wool-like PCL NPs can co-deliver multiple therapeutic agents with a single nanoparticle for combinatorial therapy.

To study drug release under extra and intracellular conditions wool-like Nil/IM PCL NPs were incubated in PBS at different pH values (pH 5.0; 6.5 and 7.4). IM was released by intracellular protease degradation of DXS and as shown in Figure 2B a low IM release percentage was observed at different pH values (about 38% at pH 5.0 after 96 h). Conversely, at lower pH, the Nil release rate was considerable faster, with about 72.42% (pH 6.5) and 91.72% (pH 5.0). As shown in Figure 2C, Nil release at pH 7.4 was slow and constant, with a release percentage of about 17.55% in 96 h. These dissimilar release behaviours of IM and Nil were due to the different polymers (DXS for IM and CH for Nil) that were used in the complexation process before sparkling and wool-like PCL NPs assembly. In particular, IM was complexed with an intracellular protease sensitive polymer, such as DXS. In this way IM was released in the cytosol compartment after degradation of backbone of DXS polymer by intracellular protease [35,36] and this allowed the drug first release (Figure 2A). On the other hand, Nil was complexed with a pH sensitive polymer, such as CH and successively mixed with a combination of sodium bicarbonate and potassium tartrate (NaHCO_3_ + KC_4_H_5_O_6_) in the core of PCL NPs. In an acid environment, such as endosome (pH 6.5) and lysosome (pH 5.0) [49], the mixture of sodium bicarbonate and potassium tartrate quickly generates CO_2_ bubbles producing large pores in the NP shell and burst release of Nil contents, allowing the second release (Figure 2A). No CO_2_ bubbles were generated from PCL NPs at pH 7.4. Synergistic achievement of both TKI molecules (IM and Nil) on sustained targeting of BCR-ABL with low therapeutic dosage can be achieved using a single stimuli responsive co-delivery system, such as wool-like IM/Nil PCL NPs.

### 3.2. Cellular Uptake and Intracellular Localization of Wool-Like PCL NPs

**Figure 3:**
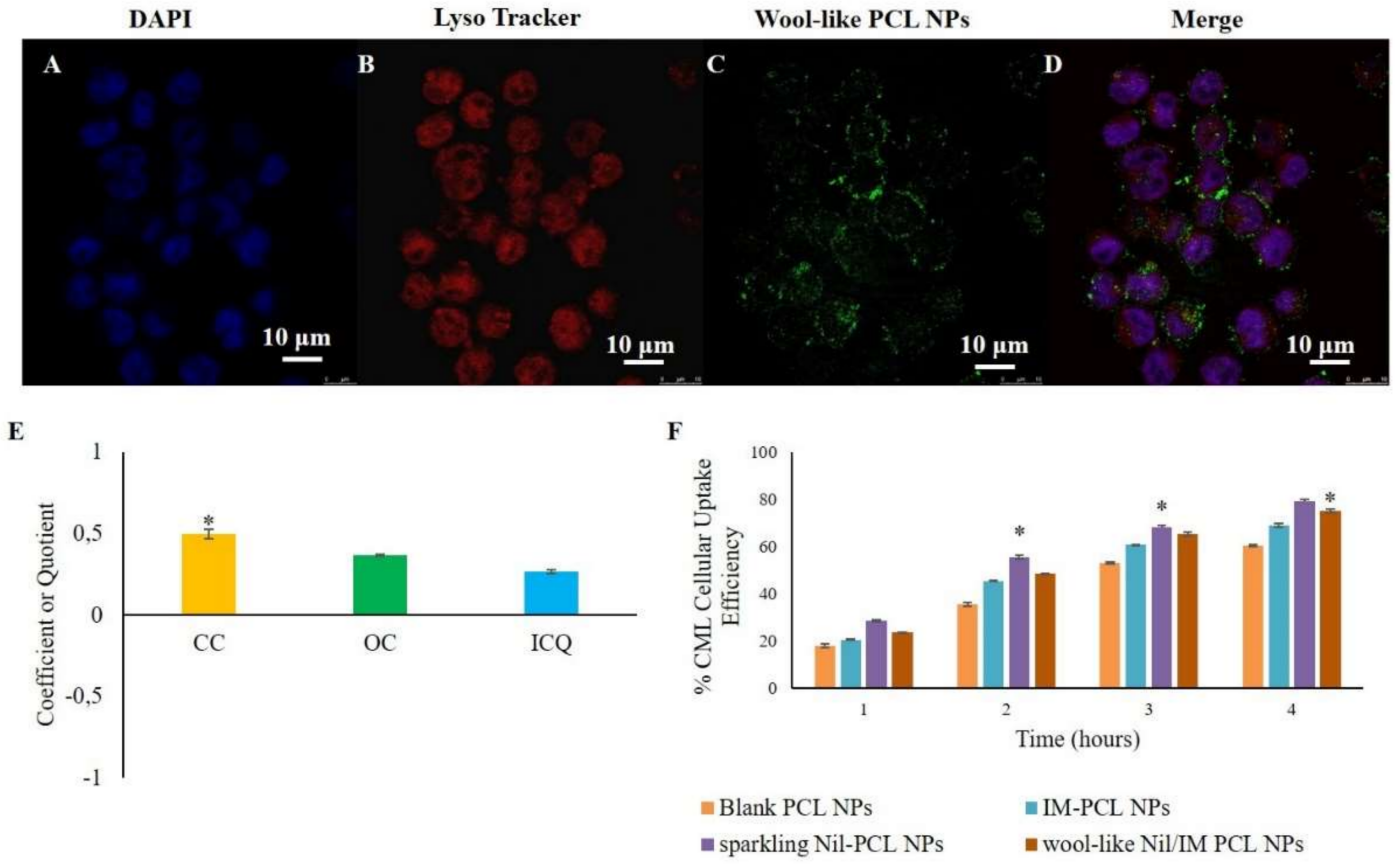
Confocal laser scanning microscopy (CLSM) images of cellular uptake and intracellular localization of wool-like DiO PCL NPs ((**C**), green) in KU812 leukemic cells after 1 h of incubation. Cell nuclei were counterstained with DAPI ((**A**), blue). Lysosomal compartment was marked with Lyso-tracker ((**B**), lysosome marker, red). The merged image (**D**) shows the co-localization of wool-like DiO PCL NPs (**C**) with Lyso-tracker (**B**). Pictures were imaged with a 63X oil immersion objective. Scale bars: 10 µm. (**E**) Quantitative co-localization analysis between wool-like DiO PCL NPs and LysoTracker for KU812 leukemic cells. For the same confocal images, the CC (coefficient of correlation), OC (overlap coefficient) and ICQ (intensity correlation quotient) were calculated and plotted. Ten different fields were randomly selected for each sample and three distinct experiments were performed; * indicates *P*-values of <0.05 for *t*-Student test. (**F**) Time-dependent cellular uptake efficiency of blank PCL NPs, IM PCL NPs, sparkling Nil loaded PCL NPs and wool-like hollow Nil/IM loaded PCL NPs by KU812 cells. Representative measurements of three independent experiments have been reported and * indicates *P*-values < 0.05 for Student’s *t*-test between different time points.

Cellular uptake and intracellular localization of fluorescent wool-like DiO PCL NPs in leukaemia KU812 cell line and healthy C13895 cells was studied by confocal laser scanning microscopy (CLSM) (Figure 3A–D and supplementary Figure S1). After 1 h of incubation, NPs appeared as fluorescent dots with a predominant distribution in the cytoplasm (Figure 3D).

Nanosystem intracellular fate is strongly affected by the entry way. Polymeric NPs internalization into the cells occur by endocytic pathways [50,51]. In this context, we have previously reported that different polymeric NPs, such as polyelectrolyte complexes (PECs) [36] and PCL NPs [40], with an average size of 250 nm were internalized with a clathrin-mediated endocytosis mechanism, because clathrin-coated invaginations on the cell membrane enroll payload with variable shape and size. On the contrary, caveolin-coated vesicles typically limits the internalization of NPs with a size of 20–40 nm [52,53]. Similar results were obtained with wool-like PCL NPs (supplementary Figure S2) with a size of 270 nm. In particular, we have observed a polarized co-localization of red clathrin (supplementary Figure S2B) and green wool-like PCL NPs (supplementary Figure S2C) in the cytoplasm after 1 h of incubation (Figure 3D). After 3 h a clathrin reorganization in the cells was clearly observed (supplementary Figure S2E–H).

Delivery system escape to endo-lysosomal is essential to improve the payload, particularly if the active agents encapsulated are sensible to lysosomal degradation [51]. Low co-localization in lysosomal compartment of green wool-like PCL NPs (merge channel of CLSM images of Figure 3D) was observed and this was confirmed by quantitative analysis between green NPs and red LysoTracker in KU812 leukemic cells (Figure 3E). The coefficient of correlation (CC, usually in the range of −1 to 1, where 1 means the perfect overlap and 0 means random distribution) between NPs and LysoTracker was around 0.498. The overlap coefficient (OC, typically in the range of 0 to 1, where 0 means no overlap and 1 means overlap) was 0.366, while the intensity correlation quotient (ICQ, in the range of −0.5 to 0.5, where 0.5 means overlap and −0.5 means random overlap) was 0.265. These quantitative analyses confirmed the low co-localization of the wool-like PCL NPs in the lysosome, associated with the modifications in the surface characteristics of wool-like PCL NPs after cellular uptake.

In the cytoplasm compartment, the intracellular protease allowed the degradation of DXS with consequent release of IM. The surface cationization of sparkling Nil-PCL NPs (4.644 mV) in the lysosomal compartment was retained to be responsible for their escape into cytoplasm through a mechanism analogous to that of working cationic lipids [54].

In addition, the cellular uptake efficacy of blank PCL NPs, IM PCL NPs, sparkling Nil loaded PCL NPs and wool-like hollow Nil/IM loaded PCL NPs was also quantitatively valuated by fluorimeter analysis after a time window of 4 h. As shown in Figure 3F, the cellular uptake was time dependent and the positive charge surface of sparkling PCL NPs revealed an enhanced cellular uptake for KU812 compared with other NP formulations that presented a negative zeta potential.

### 3.3. Combinatorial Cytotoxicity of Nil and IM in CML Cells

**Figure 4:**
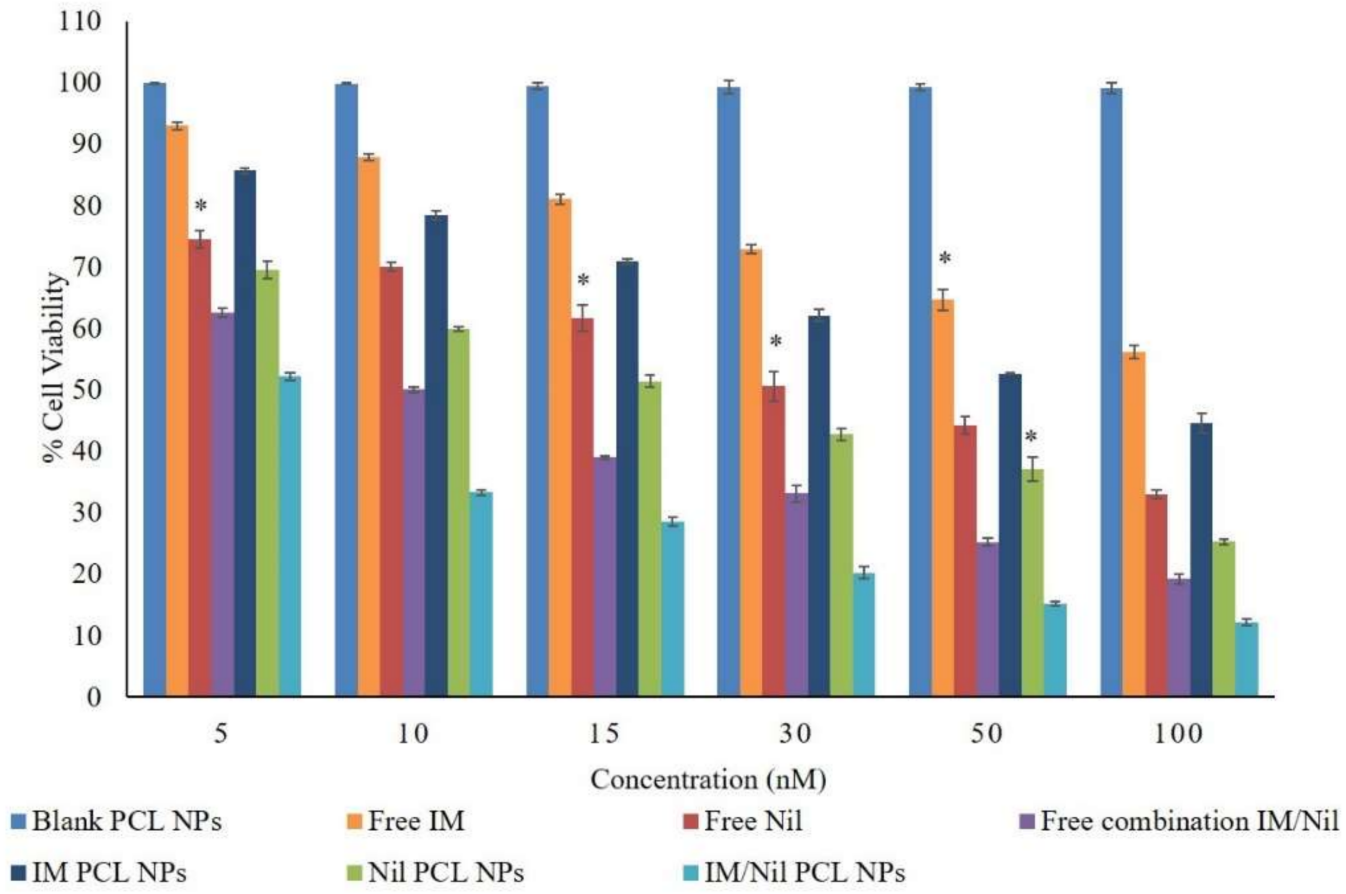
Dose dependent cytotoxicity of different concentration of free IM, free Nil, free IM/Nil (ratio 3:1) combination, IM-PCL NPs, sparkling Nil-PCL NPs and wool-like IM/Nil (ratio 3:1) PCL NPs after 48 h toward KU812 leukaemia cells. Representative measurements of three independent experiments have been reported and * indicates *P*-values < 0.05 for Student’s *t*-test between different concentrations.

In order to assess in vitro cytotoxicity of the different nanoparticle formulations, KU812 leukaemia cells were treated for 48 h with IM or Nil as a free single agent or loaded in NPs or as free combination or both loaded in wool-like PCL NPs. Free IM, free Nil, IM/Nil free combination as well as blank PCL NPs were used as controls. No adverse effects on healthy C13895 cells was detected by using freely soluble or encapsulated drugs using the IC_50_ doses (supplementary Figure S3), because healthy cells not present the oncoprotein BCR-ABL. As shown in Figure 4, the cell proliferation inhibition efficacies of all NP formulations revealed a forcefully dose-dependent effect.

Wool-like IM/Nil loaded PCL NPs showed a better proliferation inhibition effect over the free IM/Nil combination. The IC_50_ values of free IM, free Nil, free IM/Nil (ratio 3:1) combination, IM-PCL NPs, sparkling Nil-PCL NPs and wool-like IM/Nil (ratio 3:1) PCL NPs on KU812 leukaemia cells are shown in Table 1.

The IC_50_ value of free IM/Nil combination (ratio 3:1) was slightly lower to IM and Nil, used as single agents, indicating that the simple free combination achieves a mild improvement effect. This effect may be caused by fast internalization and elimination of free IM and Nil through passive diffusion by cells. Wool-like IM/Nil (ratio 3:1) PCL NPs presented an IC_50_ value much lower than free drugs and their mixture, thanks to the two-release mechanism in the cellular environment. In particular, IM was released by protease DXS degradation inside the cytosol (first release) and Nil was released, in a second time, due to the pH-sensibility of CH and formation of CO_2_ bubbles that determinate the presence of pores in the PCL shell. In this way, the IM and Nil released by wool-like PCL NPs were constantly stored inside the leukaemia cells and accomplished in playing a key role in cell death.

Synergism of IM and Nil was studied by using the combination index (*CI*) determined with isobologram equation of Chou and Talalay [45]. A synergistic effect is observed when the *CI* < 1, an additive effect is observed when *CI* = 1 and an antagonistic effect is observed when *CI* > 1. With respect to the KU812 leukaemia cells, at a ratio of IM:Nil 3:1, the *CI* was 0.83, indicating a synergistic combinatorial therapy (Table S1). In vitro cytotoxicity showed that IM and Nil released from wool-like PCL NPs can play improved anti-leukaemia effect at low concentrations.

**Figure 5:**
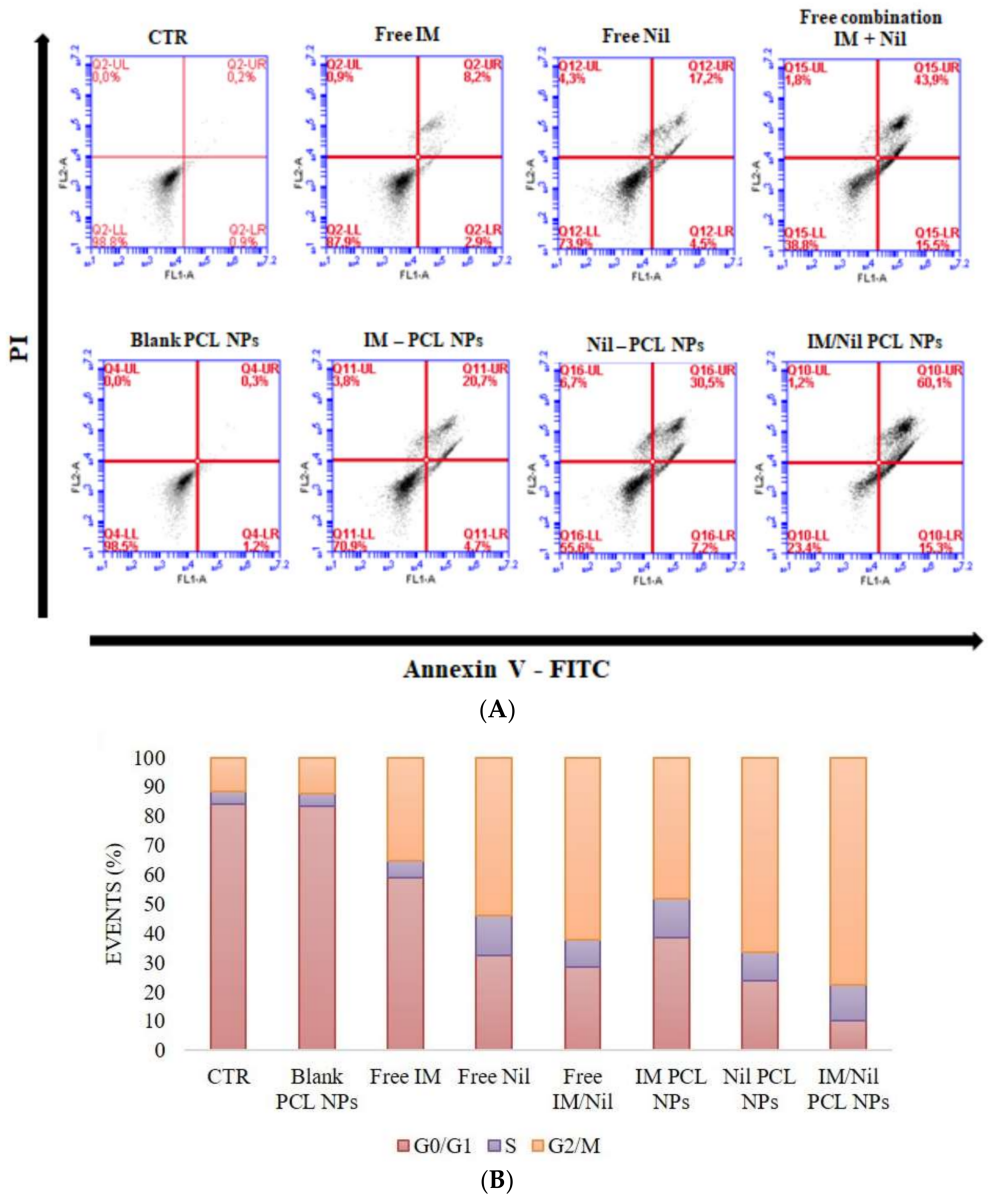
Analysis of cell apoptosis (**A**) and cell cycle (B) of KU812 leukaemia cells treated with blank PCL NPs (0.05 mg/mL), free IM (150 nM), free Nil (30 nM), free combination of IM (130 nM) and Nil (28 nM), IM-PCL NPs (70 nM), sparkling Nil-PCL NPs (18 nM), wool-like Nil/IM PCL NPs (Nil 15 nM, IM 50 nM) for 24 h.Representative measurements of three independent experiments have been reported (Student’s *t*-test, *P* < 0.05).

Cell apoptosis and cell cycle analysis were performed to endorse the combinatorial therapeutic effect of different formulations on KU812 leukaemia cells. Cell apoptosis were identified through flow cytometry using Annexin V-FITC and PI staining. Figure 5A shows flow cytometry plots of KU812 leukaemia cells after 24 h of treatment with blank PCL NPs, free IM, free Nil, free combination of IM and Nil, IM-PCL NPs, sparkling Nil-PCL NPs, wool-like Nil/IM PCL NPs. In particular, flow cytometry plot of KU812 cells treated with blank PCL NPs revealed that no change in cellular apoptosis was present (1.5%) when compared to the control (CTR, 1.1%). On the contrary, a clear improvement was detected in cellular apoptosis with a free combination of IM and Nil (59.4%) to the single free IM and Nil in solution (11.1% and 21.7%). On the other hand, pronounced cellular apoptosis was evident when KU812 cells were treated with IM/Nil co-loaded in wool-like PCL NPs (75.4%) when compared to a single drug loaded PCL NPs (25.4% for IM loaded NPs and 37.7% for Nil loaded NPs).

Figure 5B shows an evident cell cycle blocking at G2/M phase when KU812 leukaemia cells were treated with IM/Nil co-loaded in PCL NPs (77.6%) versus IM/Nil combination free in solution (62.05%).

**Figure 6:**
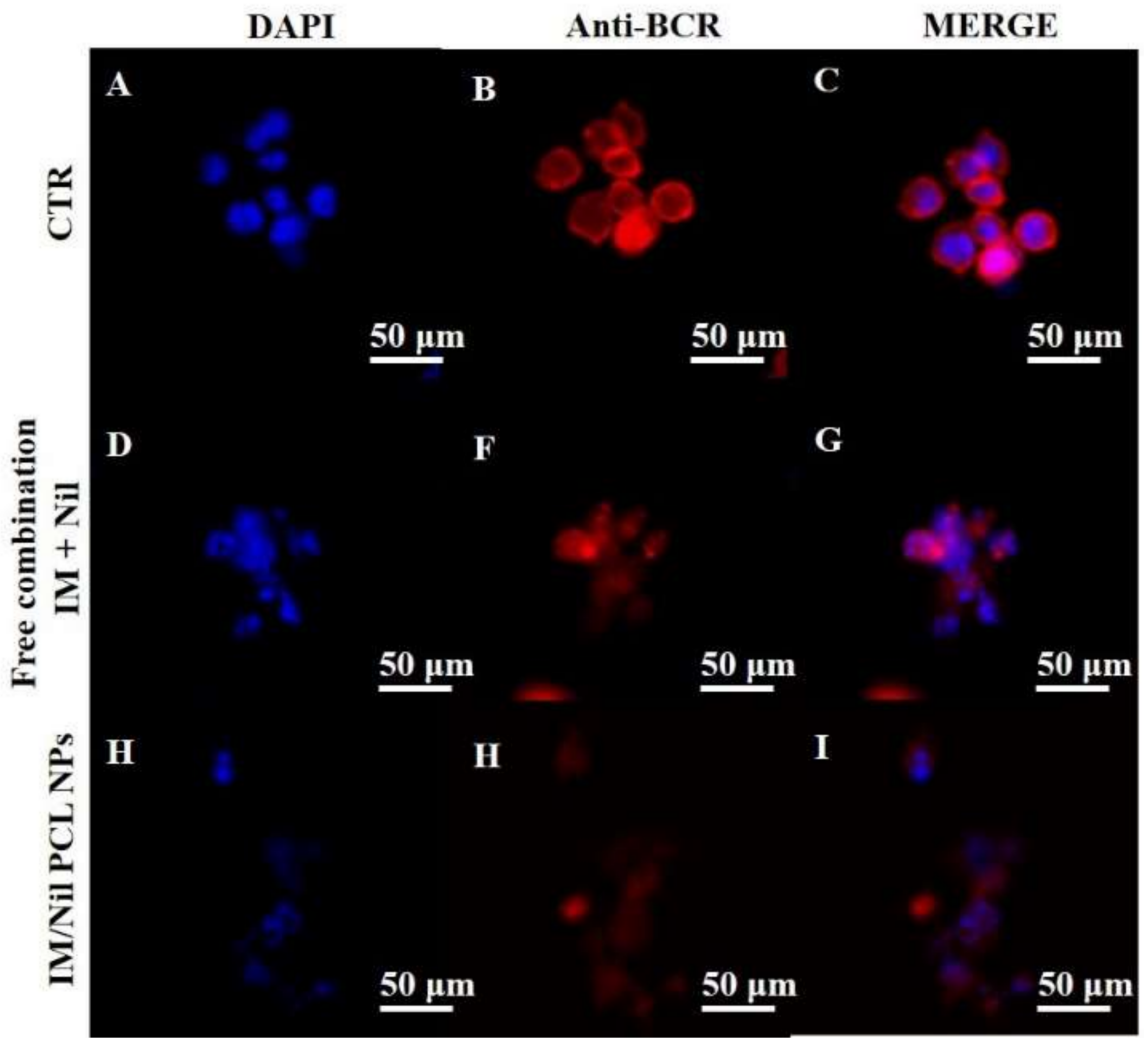
CLSM images of oncoprotein BCR-ABL cellular pattern distribution (B, F, H, red) in KU812 leukaemia cells after treatment for 24 h with free combination of IM (130 nM) and Nil (28 nM), wool-like Nil/IM PCL NPs (Nil 15 nM, IM 50 nM). Cell nuclei were counterstained with DAPI ((**A, D, H**), blue). C, G, I show merge images. Representative images of three independent experiments have been reported. 40X oil immersion objective. Scale bars: 50 µm.

Inactivation of BCR-ABL oncoprotein activity, required to promote CML cell death, was explored by confocal analysis of their cellular pattern distribution in KU812 leukaemia cells after treatment with different formulations of free drugs or loaded PCL NPs (Figure 6A–I). Control KU812 leukaemia cells (CTR) and KU812 treated with blank PCL NPs displayed a uniform distribution inside the cytoplasm of oncoprotein BCR-ABL and presented large single nuclei, round in shape (Figure 6 and supplementary Figure S4A–O). On the contrary, leukaemia cells treated with single drugs free in solution or in combination and drugs loaded in different formulation of PCL NPs showed a weak cytoplasm distribution of oncoprotein BCR-ABL. In addition, more leukaemia cells after treatment presented apoptosis characteristics, such as multinucleation, cell shrinkage, vacuolated cytoplasm. These apoptosis characteristics were more evident in leukaemia cells treated with drugs loaded in PCL NPs.

In general, a combinatorial treatment with different therapeutic agents is a hopeful approach to overcoming adverse side effects that restrict the efficacy of different drugs permitting low doses of each molecule or accessing context-specific multiple targets. Combinatorial treatment with a wool-like IM/Nil PCL NPs indicated a synergistic effect at different cellular levels when compared to single or combination of drugs free in solution or single loaded NPs. In addition, when both TKIs were encapsulated in a single PCL NPs, cytotoxicity was improved and the IC_50_ value was more reduced. Therefore, wool-like IM/Nil PCL NPs constrained KU812 leukaemia cells proliferation by a collective induction of G2/M arrest and apoptosis. Also, in the case of combinatorial leukaemia therapy, blocking the G2/M phase played a main role in cell cycle arrest, demonstrating an IM sensitiveness of the cells to Nil and increase of the Nil action on cell cycle. Cell apoptosis and cell cycle analysis were in agreement with the cytotoxicity studies, suggesting that combinatorial formulation of IM and Nil in a single nanocarrier elicited additional therapeutic effects.

## 4. Conclusions

We have developed a novel PCL nanosystem for combinatorial therapy in CML, characterized by a biodegradable pH sensitive core loaded with Nil and sodium bicarbonate and an enzymatic sensitive outer shell loaded with IM, for a simultaneous delivery of an anti-leukaemia cocktail. Clinical evidence has showed that co-administration of two or more therapeutic agents [9,10], may offer an additive/synergistic therapeutic effect taking to an eradication of more residual resistant leukemic cells. Moreover, the present work demonstrates the first example of wool-like and sparkling PCL spherical nanoparticles. Size distribution, high drug encapsulation, synchronized releasing of IM and Nil suggested that wool-like PCL NPs have a potential for combinatorial therapy for CML treatments. In vitro results positively showed that all nanomixures preserved the biological activity of loaded drugs for extensive time windows and led to a constant release of the active drug. More importantly, the combination of IM and Nil in single PCL NPs, have showed a more therapeutic efficiency at low dose respect to the single drug nanomixures. The strong inhibitory effect of drugs-loaded PCL NPs allowed an improved efficiency with a controlled release of therapeutic agent encapsulation and the double delivery of different drugs in single NPs allowed a synergistic therapeutic result. Supplementary studies are mandatory to explore the anti-leukaemia effect in vivo, the optimal dosages of both drugs with best anticancer efficiency and the application of our approach for the treatment of other tumours. We envision that our novel multiple drug delivery system has the potentially prospective to be translated into clinics in future for combination chemotherapy for fullest therapeutic outcomes.

## Figures and Tables

**Table 1:** IC_50_ values of different formulations toward KU812 leukaemia cells as measured with MTT assay.

Sample	IC_50_ IM (nM)	IC_50_ Nil (nM)
Free IM	150 nM	-
Free Nil	-	30 nM
Free IM/Nil combination	130 nM	28 nM
IM released from PCL NPs	70 nM	-
Nil released from PCL NPs	-	18 nM
IM/Nil released from PCL NPs	50 nM	15 nM

## Supplementary Materials

The following are available online at http://www.mdpi.com/1999-4923/10/2/52/s1, Figure S1: CLSM images of KU812 leukaemic cells (A,B,C) and healthy C13895 cells (D,E,F) after 3 h of incubation with wool-like DiO PCL NPs (B,E-green). Figure S2: Clathrin (red, B, F) immunofluorescence in KU812 leukaemia cells after 1 and 3 h of incubation with wool-like DiO PCL NPs (green,C,G). Cell nuclei (A,E) were counterstained with DAPI (blue). Merge images (D,H) shows co-localization of NPs with clathrin. Figure S3: MTT test for cellular viability of C13895 control cells cultured for 48 h in the absence (NT) or in the presence of free IM, free Nil, free IM/Nil combination, IM-PCL NPs, sparkling Nil-PCL NPs and wool-like IM/Nil PCL NPs to IC50 doses efficacy on KU812 cells. Figure S4: CLSM images of oncoprotein BCR-ABL cellular pattern distribution (red) in KU812 leukaemia cells after treatment for 24 h with blank PCL NPs (0.05 mg/mL), free IM (150 nM), free Nil (30 nM), IM-PCL NPs (70 nM), sparkling Nil-PCL NPs (18 nM). Cell nuclei were counterstained with DAPI (A, blue). Table S1. Combination index (*CI*) using different ratio of IM:Nil released by wool-like PCL NPs toward Ku812 cells. A synergistic effect is observed when the *CI* < 1, an additive effect is observed when *CI* = 1, and an antagonistic effect is observed when *CI* > 1.
