# Supplementary material for: Wool-Like Hollow Polymeric Nanoparticles for CML Chemo-Combinatorial Therapy
Source: Pharmaceutics. 2018 Apr 18;10(2):52. doi: 10.3390/pharmaceutics10020052 (PMC6027521; doi:10.3390/pharmaceutics10020052)
Supplement: Supplementary file 1 [file pharmaceutics-10-00052-s001.zip › pharmaceutics-290275-SI.pdf]

Supplementary Materials: Wool-Like Hollow Polymeric Nanoparticles for CML Chemo-Combinatorial Therapy

Barbara Cortese, Stefania D'Amone and Ilaria Elena Palamà

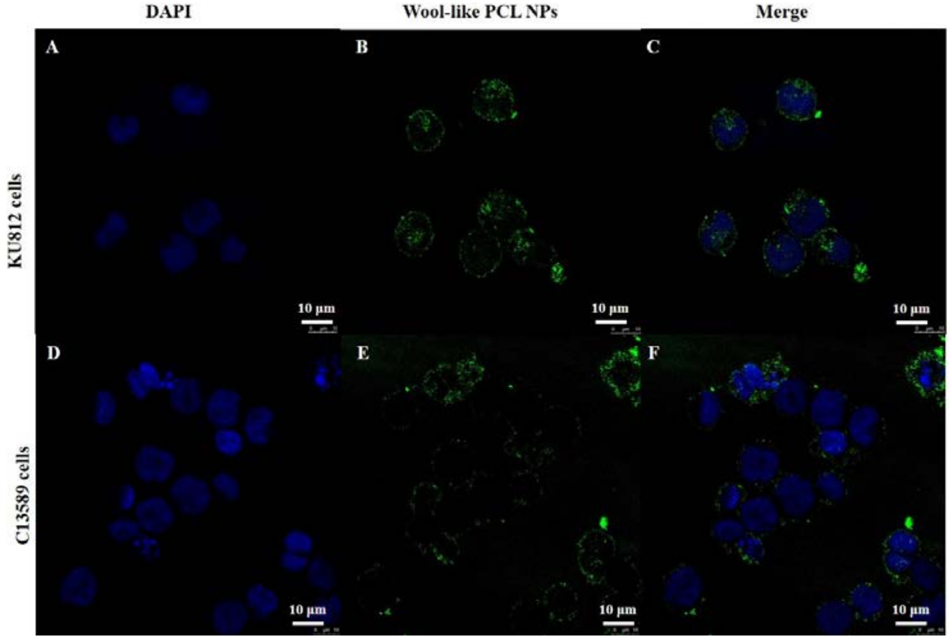

Figure S1. CLSM images of KU812 leukaemic cells (**A**, **B**, **C**) and healthy C13895 cells (**D**, **E**, **F**) after 3 hours of incubation with wool-like DiO PCL NPs (B,E-green). Cell nuclei were counterstained with DAPI (blue). Figures were imaged with a 63X objective lens. Scale bars: 10 µm.

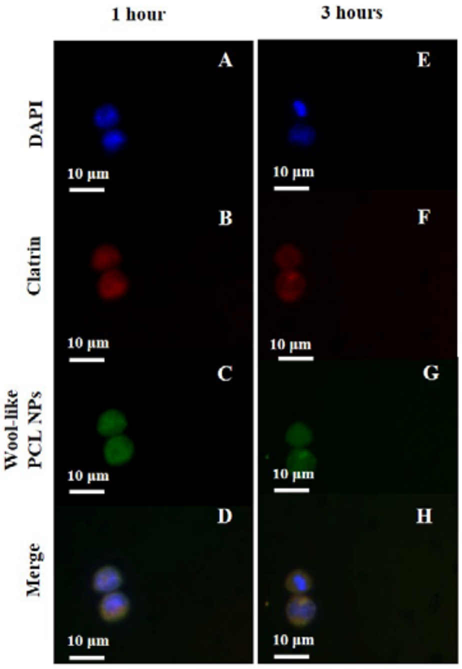

Figure S2. Clathrin (red, **B**, **F**) immunofluorescence in KU812 leukaemia cells after 1 and 3 hours of incubation with wool-like DiO PCL NPs (green,C,G). Cell nuclei (**A**, **E**) were counterstained with DAPI (blue). Merge images (**D**, **H**) shows co-localization of NPs with clathrin. Figures were imaged with a 63X objective lens. Scale bars: 10 µm.

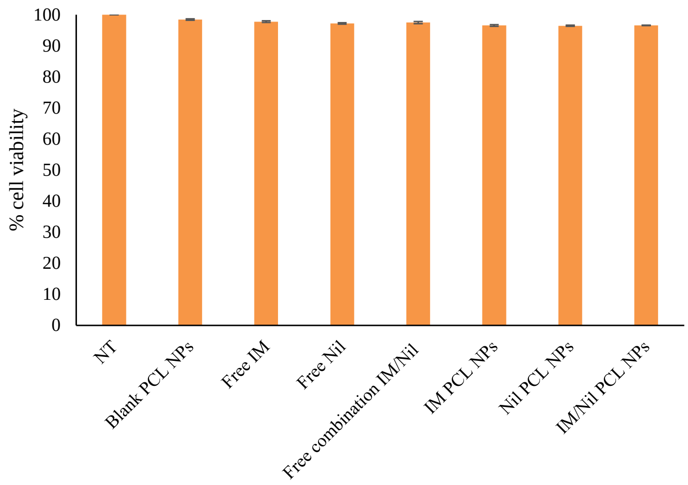

Figure S3. MTT test for cellular viability of C13895 control cells cultured for 48 hours in the absence (NT) or in the presence of free IM, free Nil, free IM/Nil combination, IM-PCL NPs, sparkling Nil-PCL NPs and wool-like IM/Nil PCL NPs to IC_{50} doses efficacy on KU812 cells. Representative measurements of three distinct sets of data have been reported (t-*Student test, P* < *0.05*).

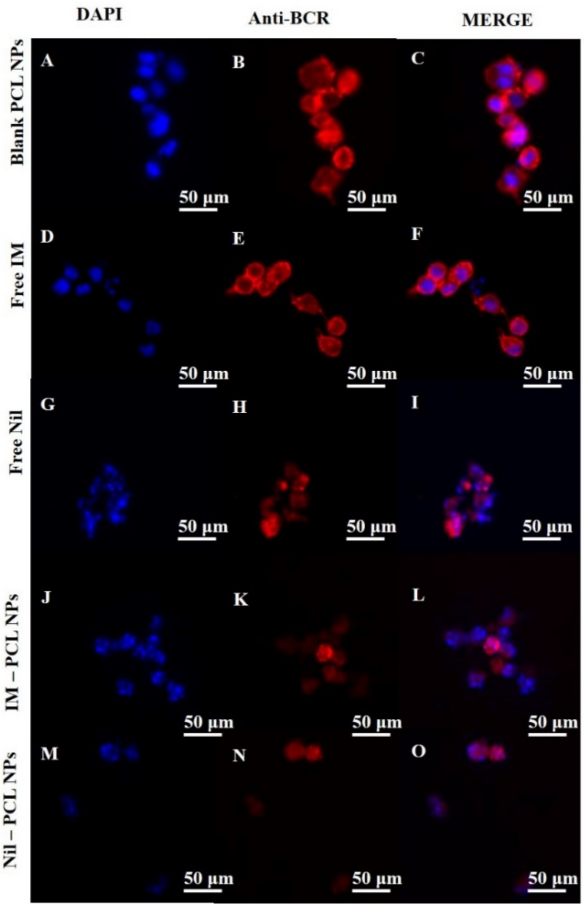

Figure S4. CLSM images of oncoprotein BCR-ABL cellular pattern distribution (B, E, H, K, N, red) in KU812 leukaemia cells after treatment for 24 hours with blank PCL NPs (0.05 mg/mL), free IM (150 nM), free Nil (30 nM), IM-PCL NPs (70 nM), sparkling Nil-PCL NPs (18 nM). Cell nuclei were counterstained with DAPI (**A**, D, G, J, M, blue). C, F, I, L, O show merge images. Representative images of three independent experiments have been reported. Figures were imaged with a 40X objective lens. Scale bars: 50 µm.

Table S1. Combination index (CI) using different ratio of IM:Nil released by wool-like PCL NPs toward Ku812 cells. A synergistic effect is observed when the CI < 1, an additive effect is observed when CI = 1, and an antagonistic effect is observed when CI > 1.

Ratio IM:Nil	CI	Effect
1:1	0.26	Synergistic
3:1	0.83	Synergistic
5:1	2.16	Antagonist
1:3	1.6	Antagonist
1:5	2.6	Antagonist
